# Integration of P, S, Fe, and Zn nutrition signals in *Arabidopsis thaliana*: potential involvement of PHOSPHATE STARVATION RESPONSE 1 (PHR1)

**DOI:** 10.3389/fpls.2015.00290

**Published:** 2015-04-28

**Authors:** Jean-François Briat, Hatem Rouached, Nicolas Tissot, Frédéric Gaymard, Christian Dubos

**Affiliations:** Biochimie et Physiologie Moléculaire des Plantes, Centre National de la Recherche Scientifique – Institut National de la Recherche Agronomique – Université Montpellier 2Montpellier, France

**Keywords:** PHR1, mineral homeostasis, phosphate, zinc, iron, sulfate, crosstalks, integration

## Abstract

Phosphate and sulfate are essential macro-elements for plant growth and development, and deficiencies in these mineral elements alter many metabolic functions. Nutritional constraints are not restricted to macro-elements. Essential metals such as zinc and iron have their homeostasis strictly genetically controlled, and deficiency or excess of these micro-elements can generate major physiological disorders, also impacting plant growth and development. Phosphate and sulfate on one hand, and zinc and iron on the other hand, are known to interact. These interactions have been partly described at the molecular and physiological levels, and are reviewed here. Furthermore the two macro-elements phosphate and sulfate not only interact between themselves but also influence zinc and iron nutrition. These intricated nutritional cross-talks are presented. The responses of plants to phosphorus, sulfur, zinc, or iron deficiencies have been widely studied considering each element separately, and some molecular actors of these regulations have been characterized in detail. Although some scarce reports have started to examine the interaction of these mineral elements two by two, a more complex analysis of the interactions and cross-talks between the signaling pathways integrating the homeostasis of these various elements is still lacking. However, a MYB-like transcription factor, PHOSPHATE STARVATION RESPONSE 1, emerges as a common regulator of phosphate, sulfate, zinc, and iron homeostasis, and its role as a potential general integrator for the control of mineral nutrition is discussed.

## Introduction

Among environmental constraints, mineral nutrition plays a key role for plant growth and development. Variations in soil nutrient composition and availability are the rule and plants have evolved mechanisms to cope with conditions ranging from extreme deficiency to toxicity due to excess. Plant breeding was oriented these last 50 years to provide crops to modern agriculture with high intrinsic growth rates and yields, under the condition that mineral nutrition was not limiting. Such condition was obtained by the massive use of fertilizers, in particular considering nitrogen (N), phosphorous (P), and potassium (K) (López-Arredondo et al., 2013). The future of agriculture will undoubtedly require to use so far uncultivated lands, some of them exhibiting unfavorable soil mineral composition, and to reduce the use of fertilizers in order to promote sustainable practices. In such a context of lower input into the environment, new cultivated plant genotypes will need to be selected in a way improving their mineral use efficiency. Reaching such a goal would be facilitated by a knowledge-based approach rooted in the understanding of how plants sense and signal changes in the availability of nutrients (López-Arredondo et al., 2013).

A wealth of knowledge was obtained these last 20 years on the physiological and morphological adaptation of plants in response to variations in availability of a given mineral element (Maathuis, 2009; Krouk et al., 2011; Gruber et al., 2013; Briat et al., 2014; Giehl and von Wirén, 2014). Genes encoding proteins involved in uptake, translocation, assimilation, and storage of macro and micro-elements have been characterized and the regulation of their expression in response to mineral status has started to be elucidated (Schachtman and Shin, 2007; Giehl et al., 2009; Gojon et al., 2009; Liu et al., 2009; Pilon et al., 2009; Hindt and Guerinot, 2012; Vigani et al., 2013). More recently multi-level studies integrating transcriptome to metabolome and to enzyme activities data enabled to begin to understand how plants reprogram various metabolic pathways in response to removal and/or resupply of mineral nutrients. It gives an insight into how plants integrate metabolism adaptation to mineral nutrition deficiency to growth (Amtmann and Armengaud, 2009; Kellermeier et al., 2014). However, it is well known that interactions between nutrients for uptake can cause imbalances if one of them is deficient or in excess (Marschner, 1995; Rouached et al., 2010). Multi-level interactions between the various mineral elements need therefore to be studied in order to understand how the different sensing and signaling pathways activated in response to changes in availability of one element are coordinately integrated with the ones of other elements.

In such a context, the principal aim of this paper is to review interactions between phosphorus (P) and sulfur (S) on one hand, and between zinc (Zn) and iron (Fe) on the other hand. In addition, phosphate (Pi) and sulfate (SO_4_) not only interact between themselves but also influence Zn and Fe nutrition, and these intricated nutritional cross-talks are presented, pointing out the emerging role of transcription factors (TFs) belonging to the MYB family.

## The MYB Family of Transcription Factors and its Role in Abiotic Stress Responses

Due to their sessile nature plants must face and adapt to a variety of biotic (e.g., bacteria, fungi, etc.) and abiotic (e.g., cold, drought, etc.) stresses throughout their life cycle. As a consequence plants have evolved molecular mechanisms allowing a tight control of their growth and development. This process is complex and dynamic, and requires the coordinated expression of several thousands of genes.

Transcription factors are sequence-specific DNA binding proteins that play a key role in the control of genes expression by acting as transcriptional activators, repressors or both. TFs possess a modular structure that is characterized by two key domains, a DNA-binding domain (DBD) and a transcriptional regulatory domain. TFs have been categorized into various families on the basis of some specific amino acid signatures mostly present in their DBD (Charoensawan et al., 2010).

**FIGURE 1:**
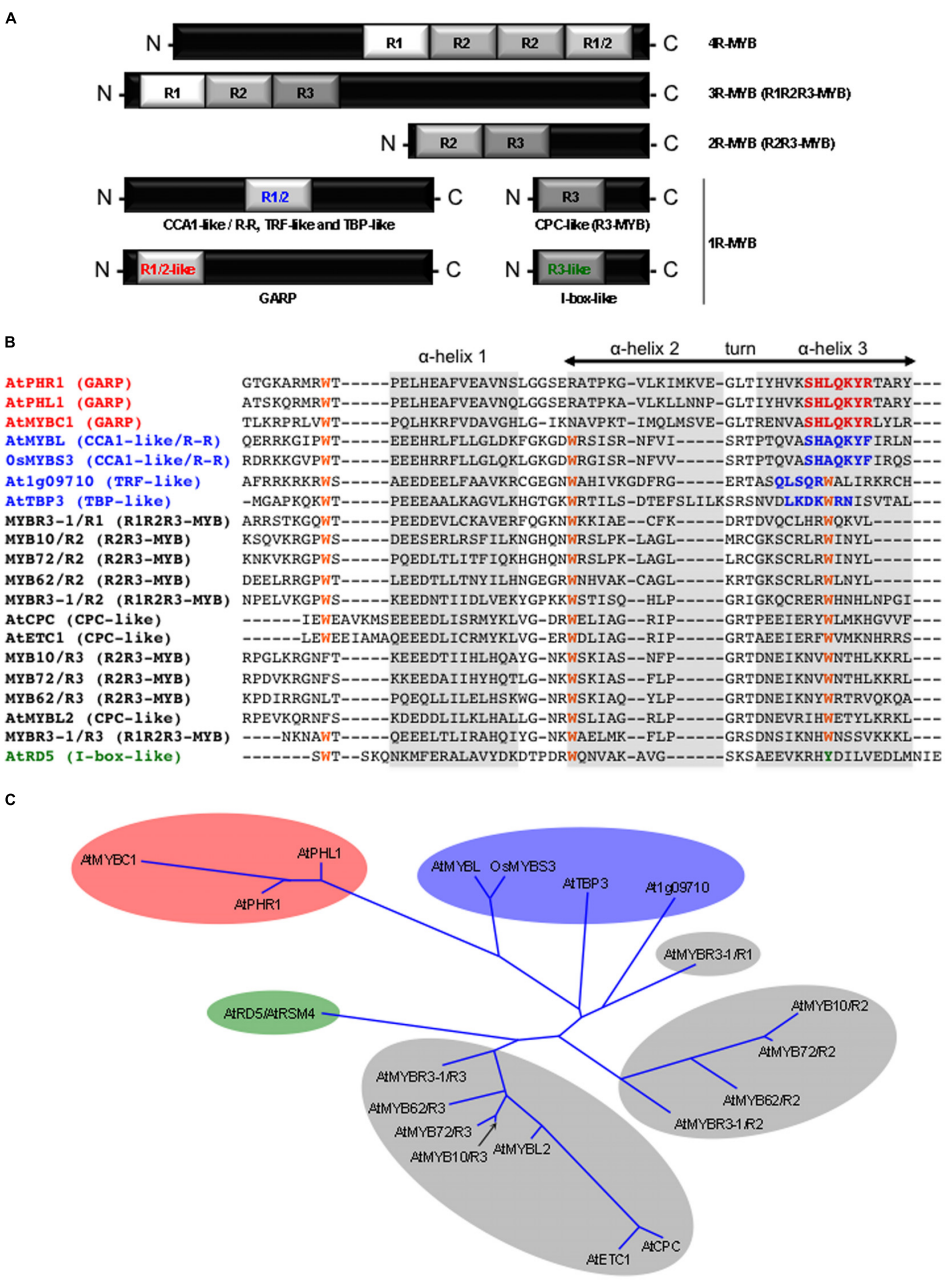
**The plant MYB protein family. (A)** Schematic representation of the different MYB protein classes based on the number (from 1 to 4) and the type (i.e., R1, R2, R3, R1/2, R1/2-like, and R3-like) of adjacent MYB repeat (R). It must be noted that the position of the R1/2 MYB repeat alongside the polypeptide chain of the proteins may vary between the different 1R-MYB subclasses that contain this domain (i.e., CCA1-like/R-R, TRF-like, and TBP-like). **(B)** Sequence alignment of MYB repeats from different classes of MYB proteins. Conserved tryptophan (W) residues are highlighted in orange. Specific amino acid signatures of the GARP, the CCA1like/R-R, TRF-like, and TBP-like, and the I-box-like 1R-MYB classes are highlighted in red, blue, and green, respectively. α-Helices are shaded in gray. The α-helix 3 is the recognition helix that makes direct contacts with DNA nitrogen bases (i.e., interacting with *cis*-regulatory sequences). Protein sequences were downloaded from TAIR (except for OsMYBS3, GenBank: accession AAN63154). **(C)** Phylogenetic relationship (unrooted tree, neighbor joining method) between the different classes of MYB repeats. The corresponding protein name is given at the extremity of each branch of the tree. The GARP, the CCA1like/R-R, TRF-like, and TBP-like, and the I-box-like repeats are shaded in red, blue and green, respectively. R1, R2, and R3 repeats are shaded in gray. (Adapted from Stracke et al., 2001, Dubos et al., 2010, and Du et al., 2013).

Among the various classes of TFs found in plants, the MYB family is one of the largest and most diverse (Riechmann et al., 2000; Dubos et al., 2010). MYB proteins are characterized by their DBD (**Figure 1**), or MYB domain. It is composed of different numbers (from 1 to 4) of imperfect repeats (R) of approximately 50 amino acids (Lipsick, 1996). Each repeat forms a helix-turn-helix (HTH) structure containing three evenly spaced tryptophan residues. These residues form a hydrophobic core playing a key role in the sequence-specific binding to DNA. The MYB gene family is divided into various groups according to the number and the type of repeat(s) found in their DBD (Stracke et al., 2001; Du et al., 2013).

To date, no biological role directly related to plant responses to abiotic stresses has been clearly reported for the 3R- and 4R-MYB proteins. 3R-MYBs are found in all eukaryotic cells where they participate to the control of the cell cycle. In contrast, the role of the plant specific 4R-MYBs remains elusive.

The R2R3-MYB class (two repeats) is the largest group of MYB-proteins exclusively found in plant species. For instance, out of the 196 MYB genes found in the *Arabidopsis thaliana* genome, 126 encode R2R3-MYB proteins (Dubos et al., 2010). R2R3-MYBs are specifically involved in the transcriptional control of plant-specific processes, including plant responses to various abiotic stresses, such as cold or drought. This TF sub-family has been extensively studied in *Arabidopsis* allowing to determine the biological role played by more than half of its members (Dubos et al., 2010). For example, AtMYB14 and AtMYB15 are involved in the plant response to cold stress (Agarwal et al., 2006; Chen et al., 2013). AtMYB60 and AtMYB96 act through the ABA signaling cascade to modulate plant response to drought, by controlling stomatal movement, and root growth and cuticular wax deposition, respectively (Cominelli et al., 2005; Seo et al., 2009, 2011). In contrast, AtMYB2 and AtMYB44/AtMYBR1 regulate the expression of their target genes in response to drought in an ABA-dependent manner (Abe et al., 2003; Jaradat et al., 2013). AtMYB88 and AtMYB124/FLP paralogs are key regulators of stomata differentiation. They have recently been shown to be involved in sensing and/or transducing salt stress (and most probably other abiotic stresses; Xie et al., 2010). AtMYB20 and AtMYB73 are also involved in salt stress tolerance, whereas AtMYB108/BOS1 displays a less specific role as it is required in the response to both pathogens and abiotic stresses, including drought, salinity, and oxidative stress (Mengiste et al., 2003; Cui et al., 2013; Kim et al., 2013).

Last, the single MYB repeat proteins forms a heterogeneous group that gather genes that can be classified into five major subgroups: the CPC-like, the CCA1-like/R-R, the I- box-like, the TRF-like, the TBP-like, and the GARP (Du et al., 2013). To date, only 1R-MYBs belonging to the CPC-like (also called R3-MYB), and CCA1-like/R-R subgroups, have been associated to plant abiotic stress responses. The *Arabidopsis* AtMYBL2 is a CPC-like MYB protein whose activity is decreased under high light stress, and which acts as a negative regulator of anthocyanin biosynthesis (which provide a natural sunscreen for plants), and (Dubos et al., 2008). Amongt the CCA1-like/R-R group of MYB proteins, OsMYBS3 has been shown to be involved in cold tolerance in rice (*Oryza sativa*), whereas AtMYBL plays a role in the *Arabidopsis* response to salt stress (Su et al., 2010; Zhang et al., 2011). AtMYBC1 that belongs to the GARP subgroup was found to be a negative regulator of freezing tolerance in *Arabidopsis* (Zhai et al., 2010).

Nutrients availability is also an important environmental factor which modulates plant growth and development, and therefore crop productivity. Consequently, deficiencies in nutrient supplies are abiotic stresses against which plants have evolved signaling cascades aiming to improve nutrient acquisition and homeostasis. Similarly to the above-mentioned abiotic stresses, MYB proteins have also been found to be involved in the plant response to nutrient deficiencies. For example, two homologous R2R3-MYB proteins (namely AtMYB10 and AtMYB72) have been shown to play a key role in improving growth under Fe-limiting conditions (van de Mortel et al., 2008; Palmer et al., 2013; Zamioudis et al., 2014). However, most of the MYB proteins identified so far as involved in the regulation of mineral nutrition were associated with Pi starvation. For example AtMYB62 participates in the response to Pi shortage (Devaiah et al., 2009), whereas AtETC1 (ENHANCER OF TRY AND CPC 1) is a CPC-like MYB TF acting to modulate root hair density under Pi limited conditions (Savage et al., 2013).

**FIGURE 2:**
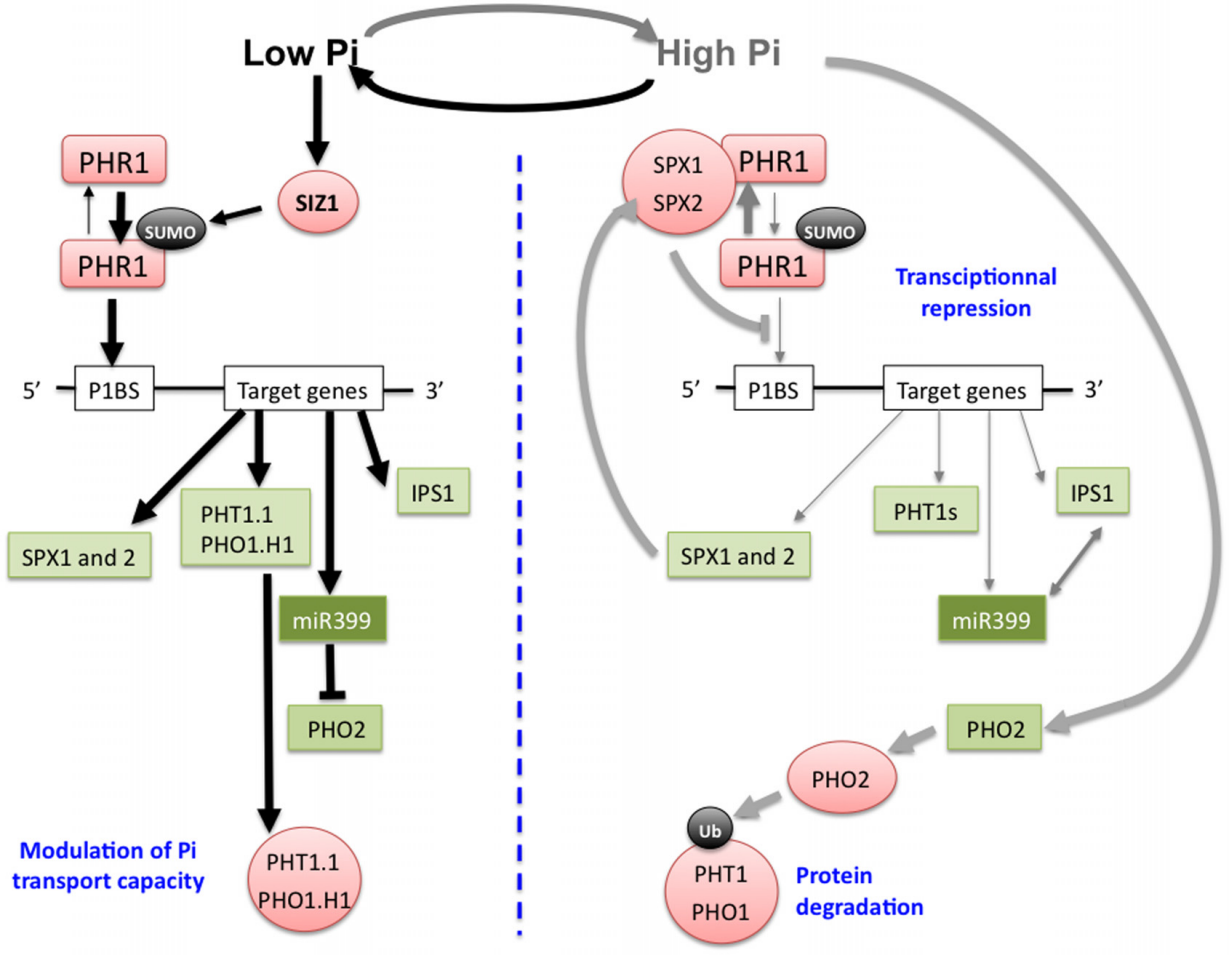
**Schematic representation of the regulatory pathways required for plant adaptation to Pi deficiency**. Under low Pi nutrition conditions **(left)** the transcriptional activation of a set of genes necessary for Pi uptake by the roots (*PHT1*, *PHO1*), occurs through binding of the transcription factor (TF) PHOSPHATE STARVATION RESPONSE 1(PHR1) to its *cis*-target present in the promoter region of these genes. Under low Pi conditions, PHR1 is sumoylated by SIZ1, and this post-translational modification is likely important for PHR1 activity because Pi-deficient regulated genes are no more induced in *siz1* mutant under this condition (Miura et al., 2005), although the mechanism of this regulation is unknown. Post-transcriptional regulators of Pi transporter proteins (PHT1.1, PHO1.H1) are also transcriptionally up-regulated through PHR1 activity under Pi-deficiency. Among them the miRNA miR399 negatively regulates the ubiquitin E2 conjugase PHO2 responsible of the ubiquitination of PHT1 and PHO1 proteins in order to target them for proteasome degradation. miR399-dependent inhibition of PHO2 can be titrated under high Pi through RNA mimicry *via* its appariement to *IPS1*, a non-coding RNA positively regulated by PHR1 under Pi deficiency. Under high Pi nutrition conditions **(right)** PHR1 target genes are transcriptionally repressed and PHO2 expression is activated promoting Pi transporters degradation. This transcriptional repression under these conditions is mediated through Pi sensing of nuclear SPX proteins which interact with PHR1 via their SPX domain in Pi-dependent manner in order to inhibit PHR1 binding to its P1BS *cis*-acting sequence found in the promoter region of Pi responsive genes. Green: transcripts, red: proteins, black: post-translational modifications, arrows thickness is proportional to the strength of the considered flux.

PHOSPHATE STARVATION RESPONSE 1 (PHR1) and PHR1-Like 1, (PHL1) are two homologous GARP MYB proteins that play a critical role in the adaptation of plant to Pi (inorganic phosphate) deficiency. Originally, PHR1 was first identified as a regulator of Pi nutrition in *Chlamydomonas reinhardtii*, and named PSR1. It is required for the transcriptional induction of Pi-deficiency responsive genes in this green algae (Shimogawara et al., 1999; Wykoff et al., 1999). A genetic screen enabled to identify a PSR1 ortholog in *Arabidopsis*, AtPHR1 (Rubio et al., 2001). Several years later a redundant gene to *AtPHR1* was characterized and named *AtPHL1* (Bustos et al., 2010). These two TFs are able to interact, and they recognize the same *cis* element located in the promoter sequence of their target genes. A consensus sequence of this element, called *PHR1 Binding Site* (*P1BS*), has been defined as 5′-*GNATATNC*-3′ (Rubio et al., 2001; Bustos et al., 2010; **Figure 2**). Transcriptome analysis revealed that most of the genes induced in response to Pi deficiency lost this ability in *phr1 phl1* double mutant plants. Furthermore, the frequency of occurrence of the *P1BS* element is much higher in the promoter region of genes induced in response to Pi deficiency than in others (Bustos et al., 2010). Interestingly, two *Arabidopsis* PHR1 orthologs, *OsPHR1,* and *OsPHR2*, have been characterized in rice (Zhou et al., 2008), and functional *P1BS cis*-elements have been reported in barley (Schünmann et al., 2004a,b). These data indicate that the regulatory pathway involving PHR1 to activate the expression of some genes in response to Pi starvation is likely to be conserved between monocotyledonous and dicotyledonous plants. It is noteworthy that a recent study has pointed out that the expression of *AtFer1*, a key gene involved in Fe homeostasis, was transcriptionally regulated by AtPHR1 and AtPHL1, providing a direct molecular link between Fe and Pi homeostasis (Bournier et al., 2013, and see below the “PHR1 involvement in Pi and Fe homeostasis interactions” section).

## PHR1 and the Control of Pi Nutrition

P is an essential macronutrient required by living organisms. It is found in essential biological molecules including nucleic acids, ATP (a major energy carrier) and phospholipids. P is intimately linked with energy metabolism, the production of numerous metabolic intermediates, and the post-translational modification of proteins (a key parameter in signal transduction cascades; Poirier and Bucher, 2002). Its deficiency has deleterious effects on plant growth and development, evidenced by a strong decrease in shoot growth, and by the accumulation of anthocyanins. Plants acquire P by their roots as inorganic Pi. Pi concentration is heterogeneous in the soil, and often very low at the root/soil interface (Poirier and Bucher, 2002). Root architecture responds to Pi deficiency by inhibiting primary root growth and by increasing lateral root density. This is an adaptive strategy to explore more soil volume, resulting in an increased Pi uptake capacity of the plants. After having crossed the plasma membrane of epidermal and cortical root cells, Pi is distributed throughout the plant under the control of a cascade of Pi transporters belonging to the PHT and PHO1 families. For an extensive review of the Pi transporter gene family, readers are referred to Poirier and Bucher (2002), and to Nussaume et al. (2011). Plant adaptation to Pi deficiency involves wide changes in gene expression. It implicates several TFs. Few of them have been identified including WRK75, ZAT6, bHLH32, MYB62, and PHR1 (Yi et al., 2005; Chen et al., 2007; Devaiah et al., 2007a,b, 2009; Svistoonoff et al., 2007; Ticconi et al., 2009). So far, the major regulations of the expression of numerous Pi deficiency-induced genes was attributed to PHR1, *via* the PHR1-PHO2–miRNA399 pathway described below (**Figure 2**). In addition to this pathway, plants respond to Pi deficiency through pathways involving phytohormones and various metabolites (Buchner et al., 2004). Among phytohormones, cytokinins suppress the up-regulation of several genes in response to Pi deficiency, including Pi uptake transporters. It requires the cytokinin receptor CYTOKININ RESPONSE 1/WOODEN LEG/*ARABIDOPSIS* HISTIDINE KINASE 4 (CRE1/WOL/AHK4) pathway (Maruyama-Nakashita et al., 2004; Franco-Zorrilla et al., 2005). Among the metabolites, carbohydrates are involved in the Pi deficiency response. The expression of Pi transporters is sensitive to the carbon status of the plant, either upstream or downstream of the hexokinase (HXK) activity in glycolysis (Lejay et al., 2008).

At a molecular level Pi deficiency is regulated both at the transcriptional and post-transcriptional levels. The major actors coordinating these various regulations are PHR1 and PHL1, two TFs likely conserved amongst flowering plants.

Indeed, sensing of the Pi status of the plant is likely conserved between mono- and dicotyledonous plants, as recently reported (Wang et al., 2014a). Expression of *AtPHR1* and of its rice orthologous gene *OsPHR2*, are not responsive to Pi, raising the question of how plants sense the intra-cellular variations of Pi concentrations. In this context, Wang et al. (2014a) recently reported that rice OsSPX1 and 2, which are nuclear proteins whose expression is itself activated by OsPHR2 under low Pi conditions, interact with OsPHR2 by their SPX domain in a Pi-dependent manner. This interaction results in the inhibition of OsPHR2 binding on its *cis*-acting *P1BS* sequence (**Figure 2**). Therefore, this mechanism constitutes a very efficient transcriptional regulatory feedback loop to fine tune the PHR1 dependent expression of Pi responsive genes, according to the intracellular fluctuations of Pi concentrations.

Among the genes transcriptionally regulated by PHR1/PHL1 in response to Pi deficiency are the *PHT1* genes (**Figure 2**). They encode plasma membrane high-affinity H^+^/Pi co-transporters (Okumura et al., 1998; Mudge et al., 2002), preferentially expressed in the root epidermal or cortical cells, and therefore directly involved in Pi acquisition (Karthikeyan et al., 2002; Mudge et al., 2002). Once Pi has entered the root, it is loaded into the xylem sap for translocation to the shoots *via* PHO1, which is specifically expressed in the pericycle (Hamburger et al., 2002). PHT1 and PHO1 transporters are also post-translationally regulated during their intracellular trafficking to the plasma membrane, and the C terminus phosphorylation of PHT1 proteins retains them at the ER upon Pi refilling (Bayle et al., 2011). During the post-ER trafficking, the ubiquitin E2 conjugase PHO2 modulates the ubiquitination status of PHT1 and PHO1 transporters in order to control their rate of degradation by the proteasome. *PHO2* expression is itself post-transcriptionally repressed by miR399, a small non-coding RNA up-regulated by Pi deficiency at the transcriptional level through PHR1/PHL1 activity (Fujii et al., 2005; Aung et al., 2006; Bari et al., 2006; Chiou et al., 2006; Liu et al., 2014; **Figure 2**). Post-translational control of Pi loading into the xylem *via* the degradation of PHO1 under Pi-sufficient conditions has also been reported to be mediated by PHO2 (Liu et al., 2012).

Phosphate Starvation Response 1 is itself regulated post-translationally in response to Pi deficiency through the action of SIZ1, a sumo E3 ligase (**Figure 2**). PHR1 can be sumoylated *in vitro* by SIZ1, and Pi-deficient regulated genes are no more induced in *siz1* mutant in response to Pi deficiency (Miura et al., 2005). This post-translational regulation of PHR1 could modify its activity, in order to modulate the transcriptional activation of its target genes, among which the *IPS1* gene (Rubio et al., 2001). *IPS1* encodes a non-coding RNA whose sequence is in part complementary of the microRNA miR399, enabling post-transcriptional regulation *via* RNA mimicry (Liu et al., 2014). IPS1-miR399 matching would therefore lead to the inhibition of the miR399 RNA activity (Franco-Zorrilla et al., 2007) known to target *PHO2* (**Figure 2**). It could explain the increased expression of PHT1-8 and PHT1-9 in *pho2* mutant plants (Bari et al., 2006), and the decreased expression of *PHO2* and of the *PHT1-8* and *PHT1-9* Pi transporter genes in miR399 over-expressing transgenic lines (Aung et al., 2006). The involvement of *PHT1-8* and *PHT1-9* in Pi uptake (Remy et al., 2012) and/or in Pi root-to-shoot translocation (Lapis-Gaza et al., 2014) has been reported. More recently an iTRAQ based quantitative membrane proteomic approach was used to search for components downstream of PHO2. It enables to show that PHO2 mediates the degradation of PHT1 proteins by interacting in the post-endoplasmic reticulum compartments where ubiquitination of endomembrane-localized PHT1-1 occurs (Huang et al., 2013). Finally the systemic effect of miR399 was evidenced by grafting experiments. It demonstrated the transport of this small RNA from leaves to root within the phloem sap, explaining why specific overexpression of the miR399 in leaves led to decrease the expression of *PHO2* in roots (Pant et al., 2008).

## Pi, S, and Their Biological Interactions

Sulfur is an essential element for plant growth and development. The major source of S for plants is inorganic sulfate (SO_4_; Leustek et al., 2000). In the cell, S is involved in different functions and aspects of plant metabolism. It is found both in reduced (amino acids, peptides, and proteins, lipoic acid, iron–sulfur clusters), and in oxidized (polysaccharides, lipids, and sulfonate group modifying proteins) forms (Kopriva, 2006). Thus S deficiency has profound effects on plant physiology. When challenged by SO_4_ deficiency, shoot growth is affected, resulting in a decrease in the total biomass. Modulation of root system architecture has also been observed in response to S limitation (Osmont et al., 2007). The primary root continues to grow, but the lateral roots form closer to the root tip, with an increased density. During the last two decades, a comprehensive view of SO_4_ transport in *Arabidopsis* and other plant species has emerged. Numerous “*SULTR*” genes encoding SO_4_ transporters for uptake as well as for inter-organs and subcellular distribution have been functionally characterized (Takahashi, 2010). The first key step for SO_4_ uptake in *Arabidopsis* is mainly carried out by the two high-affinity SO_4_ transporters SULTR1;1 and SULTR1;2 (Rouached et al., 2009). The possibility that SULTR1;2 may function as a sensor of S status or as a component of a S sensory mechanism has recently been proposed (Zhang et al., 2014).

Some key regulatory molecular mechanisms and components involved in the regulation of SO_4_ transport have been discovered, among which a regulatory pathway requiring miRNAs, including miR395 (Kawashima et al., 2009). This microRNA contributes to the regulation of SO_4_ root-to-shoot transfer involving the *SULTR2;1* gene. The transcript abundance of this gene is strongly decreased under deficient S conditions (Takahashi et al., 1997, 2000). It can be explained by the increased level of miR395 under such conditions (Kawashima et al., 2009), leading to a strictly restricted expression of the *SULTR2;1* gene in the xylem (Kawashima et al., 2009). miR395 acts downstream of the TF SULFUR LIMITATION 1 (SLIM1), also known as ETHYLENE-INSENSITIVE3-LIKE 3 (EIL3; Maruyama-Nakashita et al., 2006; Kawashima et al., 2009).

Plants have evolved tightly controlled mechanisms allowing the coordination of the S transport and homeostasis with photosynthesis and the carbon status, in a similar manner to Pi transport system (Lejay et al., 2008). In addition to these mechanistic similarities in the regulation of the Pi and SO_4_ transport systems, plants have developed coordinated and tightly controlled mechanisms to maintain intracellular homeostasis of both elements in response to their external availability. It has been reported that plant cells operate a rapid replacement of sulfolipids by phospholipids under S deficiency, and the replacement of phospholipids by sulfolipids during Pi deficiency (Essigmann et al., 1998; Hartel et al., 1998; Yu et al., 2002; Sugimoto et al., 2007). Such a metabolic switch attests the P/S nutritional interdependency. Interestingly, two genes necessary for the replacement of phospholipids by sulfolipids in Pi-deficient plants, *SQD1* and *SQD2*, contain a PHR1 binding sequence (*P1BS*) in their promoter, and are up-regulated by Pi deficiency in a PHR1-dependant manner (Franco-Zorrilla et al., 2004; Stefanovic et al., 2007). The accumulation of SO_4_ and Pi was affected in *Arabidopsis* lines characterized by a very low inositol-6-phosphate (phytic acid, PA) content (Belgaroui et al., 2014). The expression of genes involved in the SO_4_ and Pi transport or signaling was altered in these low PA mutants. PA emerged thus as a component of the co-regulation of SO_4_ and Pi homeostasis. Hsieh et al. (2009) reported that the increased accumulation of miR395, known to be up-regulated by SO_4_ starvation, is suppressed in Pi-deficient plants. It could be therefore a mean to increase the S translocation from root to shoot by *SULTR2;1*, enhancing thus the sulfolipid biosynthesis in replacement of phospholipids under Pi deficiency. Evidences for co-regulation of Pi and S signaling pathways are starting to emerge from recent data. Rouached et al. (2011a) reported that the SO_4_ concentration increases in roots and decreases in shoots of *Arabidopsis* Pi-deficient plants. These results indicate an adaptive regulation of the SO_4_ transport process upon Pi deficiency in plants, and particularly the inter-organ SO_4_ distribution. Interestingly, PHR1 is required for this process to take place, likely through its positive regulatory role on *SULTR1;3* expression (Rouached et al., 2011a). PHR1 has also a negative effect on the expression of *SULTR2;1* and *SULTR3;4* in response to Pi deficiency (Rouached et al., 2011a). Noteworthy, such a function is also preserved in *Chlamydomonas reinhardtii via* the PHR1 ortholog PSR1 (Moseley et al., 2009). An integrative model for the regulation of the expression of genes involved in intracellular and inter-organ SO_4_ transport under Pi deficiency in *Arabidopsis* has been proposed, in which PHR1 plays an integrative role (Rouached, 2011). Considered together, these data reveals an unsuspected level of complexity and interconnection in the regulation of SO_4_ and Pi homeostasis in plants.

## Fe, Zn, and Their Biological Interactions

Zn is an essential microelement for cell life. It is the only metal represented in all six classes of enzymes: oxidoreductases, transferases, hydrolases, lyases, isomerases, and ligases (Coleman, 1998). It also plays a structural role in regulatory proteins (Berg and Shi, 1996). Despite its essentiality, high concentrations of Zn can be toxic for the cell, and cause oxidative stress, a decrease in accumulation of ATP, disintegration of cell organelles, and biogenesis of vacuoles (Sresty and Madhava Rao, 1999; Xu et al., 2013).

Zn deficient plants exhibit deformed and chlorotic leaves, and interveinal necrosis, leading to decrease biomass production. Transport across the plasma membrane is achieved by transporters belonging to the ZIP (ZRT, IRT-like protein) family (Mäser et al., 2001) such as AtIRT1 (Vert et al., 2002; Barberon et al., 2011). A passive Zn influx can also occur through the depolarization-activated non-selective cation channel (DA-NSCC; Piñeros and Kochian, 2003), or by the voltage-independent NSCCs (VI-NSCCs; Demidchik and Maathuis, 2007). Zn loading into the xylem sap involves plasma membrane transporters which are members of the P1B-ATPases family, namely AtHMA2, and AtHMA4 (Hussain et al., 2004; Verret et al., 2004). For further information on the Zn transport in plants, readers are referred to Sinclair and Krämer (2012). So far, how plants sense and transmit the signal of Zn deficiency remains poorly understood.

Fe, as Zn, is essential. It is also potentially toxic when in excess because of its reactivity with oxygen which catalyzes the formation of free radicals able to oxidize organic molecules, ultimately leading to cell death (Briat et al., 2010b). Fe is a key element to ensure the electron flow through the PSII-b6f/Rieske-PSI complex. It is therefore essential for CO_2_ fixation by the photosynthesis process. Indeed it has been well documented that Fe is a limiting factor for biomass production, as well as for the quality of plant products (Briat et al., 2014). Fe enters the plant *via* the roots, from where it is distributed within the plant. According to the plant family considered – i.e., *graminacea* plants *versus* other plants – two mechanisms prevail for mining Fe from the soil solution. They involve, respectively, chelation of the ferric iron (Fe^3+^) by small organic molecules, among which methionine derivatives constitute the mugineic acid (MAs) family, or its reduction in its ferrous form (Fe^2+^) prior to transport across the plasma membrane of root epidermal cells (Curie et al., 2009; Morrissey and Guerinot, 2009; Conte and Walker, 2011). This transport is achieved by transmembrane proteins exhibiting transporter activities, such as YS1 responsible of the transport of Fe^3+^-MAs complexes in *graminacea*, or IRT1 responsible of the root uptake of Fe^2+^ by non-*graminacea* plants (Curie et al., 2001; Vert et al., 2002). Many other transporters, as well as soluble proteins, responsible for Fe long distance distribution, sub-cellular compartmentation, and storage have also been characterized this last decade (Morrissey and Guerinot, 2009; Briat et al., 2010a; Kobayashi and Nishizawa, 2012). The expression of these genes encoding proteins responsible of Fe homeostasis at the whole plant level is precisely regulated through integrated pathways modulated by the sensing of the Fe status of the plant. These controls mainly operate at the transcriptional level *via* a netwotk of TFs, most of them belonging to the bHLH family (Ivanov et al., 2012; Kobayashi and Nishizawa, 2012; Hindt and Guerinot, 2012), but also at the post-transcriptional level (Ravet et al., 2012). How plants sense their Fe status is now the new frontier in this field of research. It could be achieved, at least in part, by some TFs and/or by Hemerythrin motif-containing Really interesting new gene and Zinc-finger proteins (HRZs)/BRUTUS (BTS), that were recently identified both in rice and *Arabidopsis*, respectively (Kobayashi and Nishizawa, 2014).

Fe deficiency in *Arabidopsis* leads to the activation of expression of *IRT1*, the primary transporter responsible of root Fe uptake. *IRT1* has a weak substrate specificity and contributes therefore to the accumulation of a broad range of divalent transition metals including Zn (Vert et al., 2002; Arrivault et al., 2006; Haydon et al., 2012). Conversely, excess Zn causes physiological Fe deficiency. Early studies reported an absence of IRT1 protein in *Arabidopsis* roots from plants grown under Zn excess conditions (Connolly et al., 2002), suggesting that the known post-translational regulation of IRT1 protein levels through ubiquitin-mediated proteasomal degradation (Kerkeb et al., 2008; Barberon et al., 2011) might predominate under this condition. However, it was reported more recently that IRT1 protein in *Arabidopsis* grown under excess Zn increased in abundance, comparatively to plants grown under standard Zn nutrition conditions (Fukao et al., 2011; Shanmugam et al., 2011). IRT1 could therefore be a major contributor to Zn deficiency.

MTP3 (a member of the Cation Diffusion Facilitator family), HMA3 (belonging to the P1B-type ATPase family), and ZIF1 (a member of the Major Facilitator Superfamily transporters) are vacuolar membrane proteins required for Zn tolerance, and encoded by genes which are transcriptionaly activated in response to excess Zn or Fe deficiency (Becher et al., 2003; Arrivault et al., 2006; van de Mortel et al., 2006; Haydon and Cobbett, 2007; Haydon et al., 2012). Indeed MTP3 and HMA3 gene expression is decreased in the *fit* mutant. This mutant lacks a bHLH TF that activates root Fe deficiency responses, in particular the transcriptional up-regulation of the Fe(III)-chelate reductase FRO2 and of the Fe^2+^ transporter IRT1 (Colangelo and Guerinot, 2006). Interestingly, transcriptional regulation of the ZIF1 gene under Fe deficiency is independent of Fe deficiency-induced Zn accumulation (Arrivault et al., 2006; Haydon et al., 2012). The bHLH TF POPEYE, controlling a set of genes expressed in the stele and important for the control of Fe homeostasis was reported to directly interact with the ZIF1 promoter and to repress its transcription, although there is a concurrent net increase in ZIF1 transcript levels, under Fe deficiency (Long et al., 2010).

These results evidence the complexity of the cross-talks between the pathways at work to regulate Fe deficiency and Zn excess in order to establish an integrated response, and the necessity of additional work in the future to decipher them.

The two macro-elements Pi and S on one hand, and the two metals Zn and Fe on the other hand, do not only interact between themselves as reported above. Indeed Pi and S status of the plant can also influence Zn and Fe nutrition, and these aspects will now be reviewed below.

## S and Fe Homeostasis Interactions

From a biochemical point of view, Fe and S are known to interact for the building of Fe–S clusters, which are a major sink for Fe, and known to be essential for photosynthesis, respiration, and many cellular enzymatic reactions (Couturier et al., 2013). However, it is only recently that Fe and S interactions have been documented at physiological and molecular levels, although the cross-talks between the pathways regulating the integrated homeostasis of these two elements remain to be deciphered. It is well established that leaf Fe concentration decreases in S-deficient tomato, comparatively to S-sufficient control plants. This is consistent with the observation that the expression level of the *LeFRO1* gene encoding a root Fe^3+^-chelate reductase, the activity of this reductase, and the reduction based of ^59^Fe uptake, are decreased in response to S starvation (Zuchi et al., 2009). In agreement with these observations a strong repression of expression of the *Arabidopsis thaliana* IRT1 Fe^2+^ transporter in response to S deficiency was recently reported (Forieri et al., 2013). Reciprocally, it has been observed that Fe starvation modifies S uptake and assimilation. At a genome wide level, data mining of transcriptomes from Fe deficient *Arabidopsis* plants revealed a cluster of S-metabolism related genes (including genes encoding plasma membrane and tonoplast S transporters, and enzymes of the S assimilation pathway such as adenosine phosphosulfate reductase) co-expressed with Fe-deficient genes (Schuler et al., 2011). Also, the high affinity S transporter *SULTR1;1* mRNA abundance is 2.5-fold lower in absence of Fe (Forieri et al., 2013). However, a contrasted report was recently published concerning this last point (Paolacci et al., 2014). It indicated that the expression of most of the group 2 and 4 S transporters were up-regulated in Fe starved tomato plants. It turned out to be also the case of the SIST1;1 and SIST1;2 tomato high affinity transporters of the group 1, to which belongs the SULTR1;1 transporter from *Arabidopsis*. Resolution of these discrepancies concerning the impact of Fe deficiency on the regulation of expression of high affinity SO_4_ transporters clearly requires further work.

The interaction between Fe and S metabolisms has not only been studied in plants acquiring Fe from the soil through a reduction-based strategy as it occurs in tomato (*Solanum lycopersicum*) or *Arabidopsis*. It has also been investigated with graminaceous plants. Synthesis of Fe^3+^-chelators of the MAs family (Kobayashi and Nishizawa, 2012) and their release in the rhizosphere were reduced in barley (*Hordeum vulgare* L.) plants grown under S starvation (Kuwajima and Kawai, 1997; Astolfi et al., 2006). However, transcript abundance of the *YS1* gene encoding the Fe^3+^-MAs transporter (Curie et al., 2001) increased in response to Fe deficiency at the same level, whatever the S nutrition conditions imposed (Astolfi et al., 2012). The modifications of S metabolism occurring in response to Fe deficiency have also been investigated in durum wheat (*Triticum turgidum* L.; Ciaffi et al., 2013). These authors have shown that Fe deficiency under S sufficient nutrition conditions led to a S deficiency response at a molecular level. This response was characterized by an increase in abundance of some of the transcripts encoding enzymes of the S assimilation pathway, including APS reductase, ATP sulfurylase, sulfite reductase, and serine acetyltransferase. Furthermore, the activity of the corresponding enzymes was also found to be increased by Fe shortage. However, changes in mRNA abundance of some other genes of the S assimilation pathway, and the activity of the corresponding enzymes, were observed to be uncoupled in their response to Fe or S deprivation. In addition, the expression of the wheat *SULTR1;3* high affinity SO_4_ transporter was significantly increased in roots and shoots in response to both S or Fe shortage, with the highest expression level observed under Fe deficiency conditions. In contrast, the expression of the *SULTR1.1* transporter gene, mainly expressed in roots, was strongly induced in response to S deficiency, but unaffected by Fe deficiency (Ciaffi et al., 2013).

In conclusion, the interactions between Fe and S metabolisms are attested both in graminaceous and non-graminaceous plants. These interactions have started to be documented at a molecular level, reporting that Fe deficiency modifies the expression of genes involved in S transport and assimilation, and *vice-versa*. Nevertheless, the characterization of these interactions is still in its infancy, and more work is needed to understand the complexity of the integration of the various pathways involved. Of particular interest would be the study of a possible role of the synthesis of Fe–S cluster, and of their relative abundance in response to various nutritional stress, as driving forces of the Fe–S interactions (Couturier et al., 2013; Forieri et al., 2013).

## PHR1 Involvement in Pi and Fe Homeostasis Interactions

Clear physiological links have been established between Fe and Pi (Hirsch et al., 2006; Ward et al., 2008). The complexation of Fe by Pi in soils leads to the formation of precipitates, decreasing the availability of these two elements for plants. As a consequence, the high affinity root Fe^2+^ uptake system, which is induced by Fe deficiency, is also activated under Pi excess conditions (Ward et al., 2008). Conversely, Pi starvation promotes metal accumulation in plants, mainly aluminum, and Fe (Misson et al., 2005; Hirsch et al., 2006; Ward et al., 2008). From a developmental point of view, it is also well documented that a decrease of primary root growth under Pi deficiency is partly due to Fe toxicity (Ward et al., 2008; Ticconi et al., 2009). Once taken up by the roots, Pi is translocated to the shoots, and Fe can interact with Pi inside roots leading to a reduced Pi translocation to the shoots (Cumbus et al., 1977; Mathan and Amberger, 1977). The same kind of interaction has been described in leaves in which high Pi content favors chlorosis, even if the leaf Fe level is sufficient (Dekock et al., 1978). Finally, in seeds Fe is stored in vacuoles under the form of globoids composed in part of phytate (inositol hexakisphosphate = IP6)-Fe complexes (Lanquar et al., 2005). These observations indicate that Pi is a very efficient Fe chelator. As a consequence, changes in Pi homeostasis will strongly influence Fe availability.

At the molecular level, transcriptome analysis of Pi deficient plants revealed an increase in abundance of transcripts from Fe excess responsive genes, and reciprocally a decrease in abundance of transcripts from Fe deficiency responsive genes (Misson et al., 2005; Müller et al., 2007; Thibaud et al., 2010). In this context, the induction of expression of the *AtFER1* gene, encoding the Fe storage protein ferritin, in response to Pi deficiency was interpreted by some authors as a consequence of an increase in available Fe under such conditions (Hirsch et al., 2006). Indeed this interpretation is likely wrong since the abundance of *AtFER1* mRNA in response to Pi starvation was recently shown to be mediated by PHR1 and PHL1, through their binding to a *P1BS cis*-element found in the *AtFER1* promoter, independently of the plant Fe nutrition conditions (Bournier et al., 2013). Furthermore, the Fe-dependent IDRS *cis*-acting element present in the *AtFER1* proximal promoter (Petit et al., 2001a) is not required for Pi-deficient induction of *AtFER1* expression (Bournier et al., 2013). Moreover, *AtFER3* and *AtFER4* ferritins genes, lacking *P1BS cis*-element in their promoter and known to be induced by Fe excess (Petit et al., 2001b), have their expression unchanged by Pi starvation (Bournier et al., 2013). Finally, induction of *AtFER1* expression in response to Fe excess is not altered in *phr1* (Bournier et al., 2013) nor in *phr1xphl1* (Bustos et al., 2010) mutant plants.

At a cellular level, Fe distribution around the vessels was abnormal in *phr1x phl1* double mutant (Bournier et al., 2013), suggesting that PHR1 and PHL1 may link Fe and Pi homeostasis not only by regulating *AtFER1* gene expression, but also by having a broader regulatory function of Fe metabolism. Indeed, genome wide analysis of Pi starved wild-type (Misson et al., 2005; Müller et al., 2007; Thibaud et al., 2010) and double *phr1xphl1* mutant (Bustos et al., 2010) plants revealed other Fe homeostasis related genes such as *NAS3* (*NICOTIANAMINE SYNTHASE 3*) and *YSL8*. These genes were induced upon Pi starvation in wild type, and exhibit a decreased induction in the double mutant plants. Moreover, Fe deficiency responsive genes, including *FRO3*, *IRT1*, *IRT2*, *NAS1,* and *FRO6* were repressed upon Pi starvation in wild type and miss-regulated in the *phr1xphl1* double mutant plants. These observations strengthen a global role of PHR1 and PHL1 in the overall control of Fe homeostasis that is, by this mean, integrated to the Pi status of the plant.

## PHR1 Involvement in Pi and Zn Interactions

Pi and Zn homeostasis in plants are known to strongly interact (Loneragan et al., 1982; Cakmak and Marschner, 1986; Huang et al., 2000; Zhu et al., 2001; Misson et al., 2005; Bouain et al., 2014a; Khan et al., 2014). Nevertheless, the molecular bases of the Pi–Zn interactions remain so far poorly understood. Long-term Pi deprivation leads to Zn over-accumulation (Misson et al., 2005), and inversely Zn starvation appears to cause an over-accumulation of Pi (Loneragan et al., 1982; Huang et al., 2000; Bouain et al., 2014a; Khan et al., 2014). Transcriptome data from roots of Pi- or Zn-deficient *Arabidopsis* plants (Hammond et al., 2003; Wintz et al., 2003; Wu et al., 2003; Misson et al., 2005; van de Mortel et al., 2006; Müller et al., 2007; Bustos et al., 2010; Rouached et al., 2011b; Woo et al., 2012), could induce the expression of many Pi-related genes (van de Mortel et al., 2006), and that Pi starvation modifies the expression of genes involved in the maintenance of metal homeostasis such as Zn and Fe (Misson et al., 2005; Bustos et al., 2010). Considered together, this transcriptome data reinforce the evidence of a cross-talk between Pi and Zn signaling pathways *in planta*. Cross-analysis of these expression data showed that Pi and Zn deficiencies often act in anopposite manner on these sets of genes. Many genes induce by Zn deficiency are repressed by Pi deficiency (Bustos et al., 2010; Bouain et al., 2014b). Interestingly, it was observed that most of these genes were further repressed in the *phr1* mutant genetic background (Bustos et al., 2010; Bouain et al., 2014a). This observation further supports the implication of PHR1 in the regulation of expression of Pi-related genes under Zn limitation in *Arabidopsis* (Bouain et al., 2014b). It agrees with a report by Khan et al. (2014) showing the involvement of AtPHR1 in the cross-talk between Pi and Zn homeostasis. As aforementioned, PHR1 was already known as a major regulator of Pi deficiency signaling through its involvement in the PHR1-miR399-PHO2 regulatory pathway (Bari et al., 2006). However, this regulatory pathway is not involved in the over-accumulation of Pi in the shoot in response to Zn deficiency (Khan et al., 2014). The Zn-responsive signaling pathway in which PHR1 is involved thus remains to be elucidated. Khan et al. (2014) also identified two additional genes that are involved in the control of Pi accumulation in response to Zn deficiency, namely *PHO1* and its homolog *PHO1;H3*. PHO1 is a well-characterized root-to-shoot Pi transporter (Hamburger et al., 2002). It is likely one of the final targets of the Zn-deficiency signaling pathway. Since its level of expression is unchanged in response to Zn deficiency, one favorite hypothesis is that its activity would be regulated through a protein–protein interaction, considering that a similar mechanism involving PHO1 and PHO2 has already been reported (Liu et al., 2012). The *PHO1;H3* gene appeared to be specifically and strongly induced by Zn deficiency (Khan et al., 2014). PHO1;H3 constitutes thus an interesting entry point to study Pi–Zn crosstalk and the regulation of Pi transport under Zn limitation. A first model illustrating Pi–Zn crosstalk in plant has been proposed (Bouain et al., 2014b; Kisko et al., 2014). In the future, this model would benefit from an in depth characterization of the molecular mechanisms underlying the effect of Pi starvation on Zn nutrition, together with the effects of Zn deficiency on Pi nutrition. The identified and characterized key genes and mechanisms acting in the coordination of Pi and Zn transport and signaling in plants could help to point to specific mutants or genetic variants that could be used in breeding programs. Taken together, this knowledge should have important consequences for both basic and applied research in agronomy and should be valuable to plant biologists, agronomists and breeders.

## PHR1 as an Integrator of Multiple Nutrition Signals and Beyond

**FIGURE 3:**
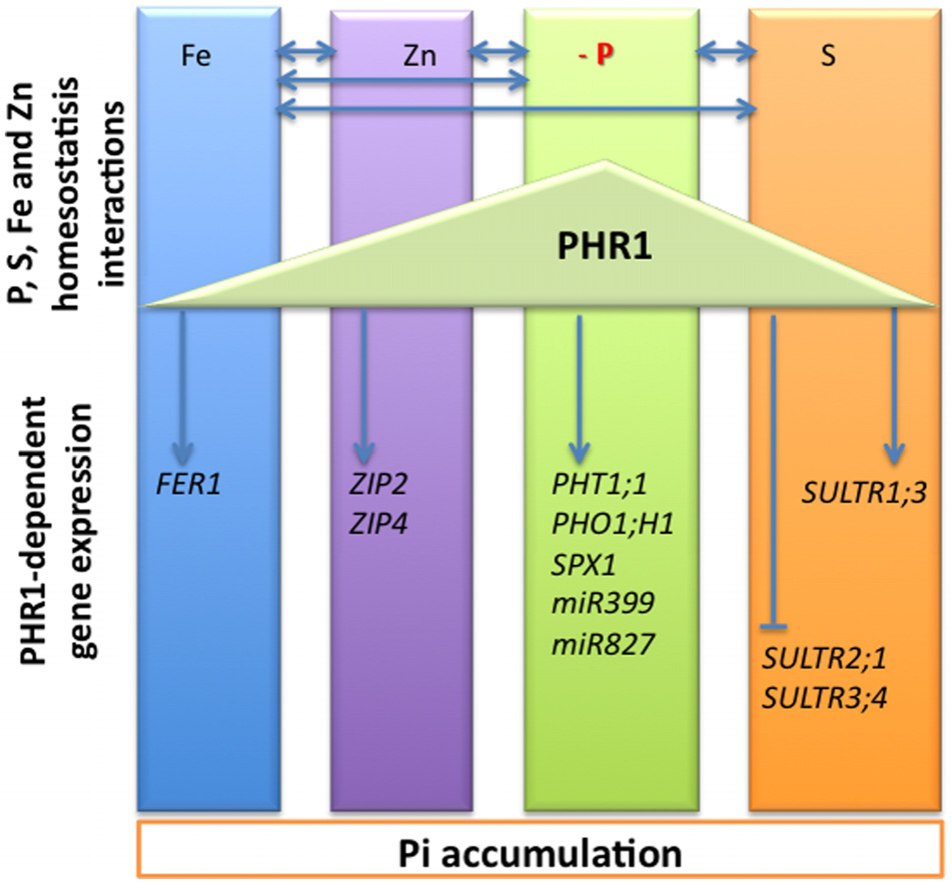
**Schematic representation of the macro- and micronutrients homeostasis crosstalks**. The interactions between phosphorus (P), sulfur (S), iron (Fe), and zinc (Zn) homeostasis are indicated by left right arrows. At a molecular level, the PHR1 transcription factor was initially identified as a key regulator of the expression of phosphate starvation induced (PSI) genes, including phosphate transporters *PHT1;1*, *PHO1;H* 1, and genes involved in phosphate deficiency sensing and signaling including *SPX1*, *miR399,* and *miR827*. PHR1 appeared also as a regulator of the expression of genes involved in sulfate transport including the sulfate transporters *SULTR1;3*, *SULTR2;1,* and *SULTR3;4*. The arrowheads and flat ended lines indicate the positive and negative effects of PHR1, respectively. The transcriptional regulation of some genes involved in maintaining Fe and Zn homeostasis has also been shown to be PHR1-dependent; it includes the *FER1* gene encoding the Fe storage protein ferritin, and the *ZIP2* and *ZIP4* genes encoding zinc transporters.

PHR1 was initially described as a major transcriptional regulator of Pi homeostasis. It activates the transcription of Pi deficiency responsive genes encoding Pi transporters (Rubio et al., 2001), as well as regulatory RNA (Fujii et al., 2005) and proteins involved in the post-transcriptional and post-translational regulation of these transporters, respectively (Aung et al., 2006; Bari et al., 2006; Liu et al., 2012; **Figure 2**). More recently, few reports indicated that PHR1 regulated also the expression of genes required for S, Fe, and Zn transport and homoeostasis (Rouached et al., 2011a; Bournier et al., 2013; Khan et al., 2014), linking the metabolism of these nutriments to Pi nutrition (**Figure 3**). PHR1 is therefore the first molecular link common to various pathways controlling mineral nutrition of both macro- and micro-elements. It can be extended to some aspects of the control of plant response to water stress, for which AtPHL1 activity is required (Elfving et al., 2011). As mentioned above, in response to Pi starvation PHR1 controls the transcription of post-transcriptional and post-translational regulators such as miR399, IPS1, and PHO2 (**Figure 2**). Whether or not these PHR1-dependent regulators play a role in the regulation of S, Fe, or Zn metabolisms is unknown, and would deserve to be investigated. Furthermore, the requirement of PHR1 has been documented in the case of two by two interactions of only some of the elements considered: Pi and S, Pi and Fe, Pi and Zn but to our knowledge no reports mentioned a direct role of PHR1 in the Fe–S or Fe–Zn interactions.

PHR1 has been the most studied regulator of Pi deficiency response, but it is known that other regulators are involved. Among them TFs including WRKY75 (Devaiah et al., 2007a), ZAT6 (Devaiah et al., 2007b), MYB62 (Devaiah et al., 2009), PTF1 (Yi et al., 2005), bHLH32 (Chen et al., 2007), and WRKY45 (Wang et al., 2014b) have been reported. Furthermore, not only additional TFs have to be considered. In addition to the well-characterized role of miR399 in the regulation of Pi homeostasis in *Arabidopsis*, other miRNAs (miRNA778, miRNA827, and miRNA2111) were reported to be specifically and strongly induced in response to Pi starvation (Fujii et al., 2005; Hsieh et al., 2009; Pant et al., 2009). The role of these miRNA in the potential cross-talks between pathways regulating homeostasis of various mineral nutriments has already been suggested for miRNA82, involved in the cross-talk between Pi-limitation and nitrate-limitation signaling pathways affecting anthocyanin synthesis (Pant et al., 2009; Liu et al., 2014). Finally, chromatin modification could also be concerned through H2A.Z histone deposition *via* the nuclear actin-related protein ARP6 in order to activate the expression of many genes related to the Pi deficiency response (Smith et al., 2010).

**FIGURE 4:**
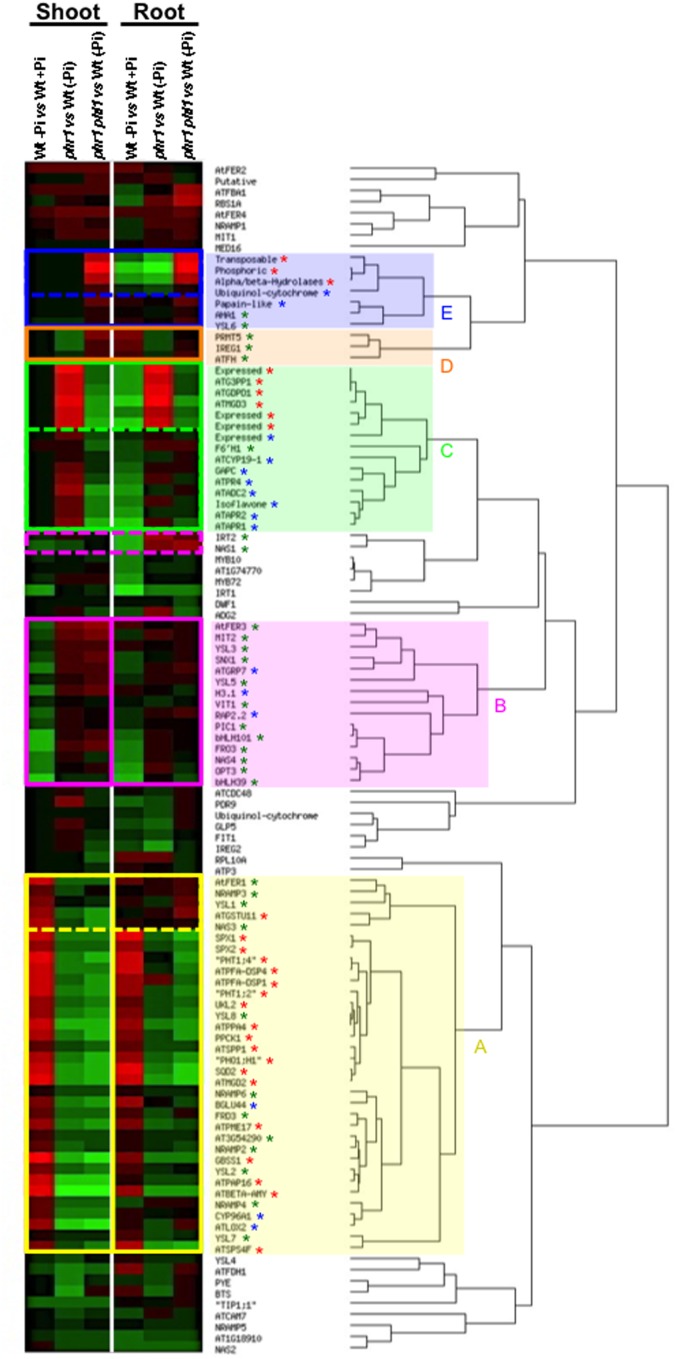
**A wider role for PHR1 and PHL1 in the regulation of plant mineral nutrition**. Transcript abundance values of genes involved in iron (Fe) homeostasis or that respond to phosphate (Pi) or sulfur (S) deficiency in wild type or *phr1* and *phr1 phl1* mutants, in the presence or absence of Pi, were selected from microarrays data (Bustos et al., 2010). These data were hierarchically clustered using EPCLUST with the default parameters (http://www.bioinf.ebc.ee/EP/EP/EPCLUST/). (**A** yellow square): Genes whose mRNA abundance is positively dependent (at least) on PHR1 activity in shoots and roots, or in shoots only (dashed lines); (**B** pink square): Genes whose mRNA abundance is negatively dependent (at least) on PHR1 activity in shoots and roots or in roots only (dashed lines); (**C** green square): revealed that Zn deficiency Genes whose mRNA abundance is positively dependent on PHR1 activity but negatively dependent on PHL1 activity both in shoots and roots or in shoots only (dashed lines). (**D** orange square): Genes whose mRNA abundance is negatively dependent on PHR1 activity but positively dependent on PHL1 activity both in shoots and roots. (**E** blue square): Genes whose mRNA abundance is negatively dependent on both PHR1 and PHL1 activities in shoots and roots or in shoots only (dashed lines). Red star, Pi deficiency response; blue star, S deficiency response; green star, Fe homeostasis.

Integration of pathways controlling two by two nutriment homeostasis has started to be documented. However a survey of transcriptome data reveals that the role played by PHR1 and PHL1 in these interactions could be wider (**Figure 4**). Indeed PHR1 and PHL1 control transcript accumulation of key genes of Fe homeostasis as well as genes whose expression is directly dependent on S or Pi availability. In consequence, a major challenge in the future will be to consider mineral nutrition as a system, and to develop tools enabling to model integrative gene networks that will take into account the availability of a maximum of macro-and micro-elements, and their interactions, at a given time.

## Conflict of Interest Statement

The authors declare that the research was conducted in the absence of any commercial or financial relationships that could be construed as a potential conflict of interest.
